# Role of white matter hyperintensity in effects of apolipoprotein E on cognitive injury

**DOI:** 10.3389/fnhum.2023.1176690

**Published:** 2023-05-19

**Authors:** Jacob Raber, Lisa C. Silbert

**Affiliations:** ^1^Departments of Behavioral Neuroscience, Neurology, and Radiation Medicine, Division of Neuroscience, ONPRC, Oregon Health & Science University, Portland, OR, United States; ^2^Department of Neurology, Oregon Alzheimer’s Disease Research Center, Oregon Health & Science University, Portland, OR, United States; ^3^Department of Neurology, Veterans Affairs Portland Health Care System, Portland, OR, United States

**Keywords:** white matter hyperintensity, white matter integrity, apolipoprotein E, magnetic resonance imaging (MRI), cognition

## Abstract

Magnetic Resonance Imaging (MRI) T2-weighted white matter hyperintensity (WMH) is a marker of small vessel cerebrovascular pathology and is of ischemic origin. The prevalence and severity of WMH is associated with cardiovascular risk factors, aging, and cognitive injury in mild cognitive impairment (MCI), vascular dementia, and Alzheimer’s disease (AD). WMH especially affects executive function, with additional effects on memory and global cognition. Apolipoprotein E (apoE) plays a role in cholesterol metabolism and neuronal repair after injury. Human and animal studies support a role for apoE in maintaining white matter integrity. In humans, there are three major human apoE isoforms, E2, E3, and E4. Human apoE isoforms differ in risk to develop AD and in association with WMH. In this Mini Review, we propose an increased focus on the role of WMH in cognitive health and cognitive injury and the likely role of apoE and apoE isoform in modulating these effects. We hypothesize that apoE and apoE isoforms play a role in modulating WMH via apoE isoform-dependent effects on oxylipins and 7-ketocholesterol, as well as amyloid related vascular injury, as seen in cerebral amyloid angiopathy.

## 1. Introduction

In humans, Magnetic Resonance Imaging (MRI) T2-weighted white matter hyperintensity (WMH), a marker of small vessel cerebrovascular pathology (Pantoni, 2010), is of ischemic origin, and regions of white matter with reduced blood flow have been shown to develop new WMH on follow-up imaging (Bernbaum et al., 2015; Promjunyakul et al., 2015). The prevalence and severity of WMH is associated with cardiovascular risk factors like hypertension (Liao et al., 1996) and with aging and cognitive injury in mild cognitive impairment (MCI) (Vettore et al., 2021), vascular dementia (Alber et al., 2019), and Alzheimer’s disease (AD) (Prins and Scheltens, 2015; Boyle et al., 2016; Lee et al., 2016; Schoemaker et al., 2022). The predominant impact of WMH on cognition is in executive function, although relationships with memory and global cognition have also been observed (Hedden et al., 2012; Debette and Markus, 2010). The concept of reserve may explain variability in the impact of WMH on cognitive function, with results from a previous study demonstrating that, for a given level of performance on neuropsychological testing, WMH were higher in those with greater cognitive and brain reserve (Brickman et al., 2011). Additional mediating factors may include age (van Dijk et al., 2008; Garnier-Crussard et al., 2020), sex (de Leeuw et al., 2001; Brickman et al., 2011), and lesion location (Mortamais et al., 2013; Lampe et al., 2019). Confluent WMH seems more associated with cognitive injury than non-confluent WMH (Kumar et al., 2020) and genetic and environmental factors modulate this relationship (Prins and Scheltens, 2015).

Apolipoprotein E (apoE) plays a role in cholesterol metabolism and neuronal repair after injury (Mahley, 1988). ApoE, a component of lipoprotein particles, binds to surface receptors like low-density lipoprotein receptor (LDLR) family members as part of this role. In addition, apoE modulates synaptic function, glucose metabolism, and cerebrovascular function (Yamazaki et al., 2019). Some effects of apoE outside the brain may ultimately affect the brain. For example, apoE is expressed in the adrenal gland and modulates the adrenal secretion of corticosterone and regulation of the hypothalamic-pituitary-adrenal axis (Raber et al., 2000). ApoE is also expressed in the gut (Niemi et al., 2002) and modulated inflammation, gastrointestinal health, and the gut microbiome composition (Tran et al., 2019). Outside the brain, most apoE is expressed in the liver and apoE secreted from the liver can also affect the brain, as illustrated in humanized liver mice (Giannisis et al., 2022; Kessler et al., 2023). In humans, there are three major human apoE isoforms, E2, E3, and E4. Compared to E3, E4 is associated with increased risk to develop cardiovascular disease and AD (Farrer et al., 1997). These E4 effects are sex- and ethnicity-dependent (Farrer et al., 1997; Turney et al., 2020). In contrast, compared to E3, E2 is associated with reduced risk of developing AD (Farrer et al., 1997). However, E2 is associated with slightly reduced LDL-cholesterol levels and in the presence of other risk factors homozygous E2 carriers can develop type III hyperlipoproteinemia, an atherogenic disorder characterized by an accumulation of remnants of triglyceride-rich lipoproteins (Khalil et al., 2021). In those cases, E2 is also associated with increased risk of developing type II Diabetes Mellitus (Santos-Ferreira et al., 2019), which especially in E4 carriers is associated with cognitive injury and AD risk (Jangra and Tople, 2022). In patients with cerebral amyloid angiopathy (CAA), a degenerative vasculopathy associated with lobar intracerebral or sulcal hemorrhage, E2 is associated with a higher incidence of intracerebral hemorrhage under oral anticoagulation (Charidimou et al., 2015; Block and Dafotakis, 2017).

In this Mini Review, we hypothesize the role of WMH in effects of apoE and apoE isoform on cognitive injury.

### 1.1. ApoE isoform and WMH in humans

In cognitively healthy adults (45–75 years of age), pathological WMH is seen in E4 homozygotes but not E4 heterozygotes, suggesting a gene-dose effect (Rojas et al., 2017; Operto et al., 2018). While aging, hypertension and cardiovascular and dementia risk scales are also associated with pathological WMH, they do not modulate the effect of E4/E4 homozygosity (Rojas et al., 2017). However, in participants of the United Kingdom Biobank (mean age: 62 years of age), E3/E4 heterozygous carriers, in addition to E4/E4 homozygous carriers, had greater WMH burden than E3/E3 carriers. Of note, E2/E4 heterozygosity was not related to greater WMH burden, consistent with a relative protective E2 effect on white matter integrity (Lyall et al., 2020). In a longitudinal study, WMH increased more over a 3-year period in E4 than non-E4 carriers, defined as having at least one e4 allele thus including heterozygous carriers (Cox et al., 2017). However, no effect of E4 carrier status (E2/E4, E3/E4, and E4/E4) on WMH burden was seen in a much smaller study (Lyall et al., 2015). In this latter study E2/E4 carriers were included, while they were not in the earlier study in which WMH was seen in both E4/E4 and E3/E4 carriers. Based on their relative protective effect of E2 versus the relative enhanced risk of E4, as compared to E3 (Farrer et al., 1997; Wisdom et al., 2011), including E2/E4 carriers can make the results harder to interpret.

Microstructural integrity disruption of the white matter, as measured by diffusion tensor imaging (DTI), is thought to be a sensitive indicator of early white matter damage that precedes macrostructural T2 WMH formation (Promjunyakul et al., 2018). Using DTI, decreased fractional anisotropy (indicating diminished WM directionality), greater radial diffusivity (indicating myelin disruption) and greater mean diffusivity (indicating greater water diffusion regardless of direction) are common indicators of diminished white matter integrity. DTI radial diffusivity seems especially increased in cognitively healthy E4/E4 carriers, suggesting a disruption in the myelin sheath rather than axonal damage (Operto et al., 2018). However, in different studies including heterozygous E4 carriers or only E3/E4 but not E2/E4 heterozygous carriers distinct alterations of white matter in E4 carriers have been reported. In 20–35 and 50–78 year-old E4 carriers, a reduction in fractional anisotropy and an increase in mean diffusivity, as compared to age-matched non E4 carriers, were reported (Heise et al., 2010). A decrease in fractional anisotropy in E4 versus non-E4 carriers were reported in people over 60 (Westlye et al., 2012) and between 49 and 79 years of age (Persson et al., 2006), respectively. However, in 21–70 year old E3/E4 versus E3/E3 carriers, so excluding E2/E4 carriers, an increase in mean and radial diffusivity were reported (Westlye et al., 2012). In general, those studies support that E4 affects white matter in an age-independent fashion. However, other studies support age-dependent E4 effects on the white matter (Ryan et al., 2011; Adluru et al., 2014). These divergent findings highlight the need for longitudinal and environmentally controlled human apoE animal studies.

In Alzheimer’s disease, WMH is more widespread in E4 carriers (E2/E4, E3/E4, and E4/E4) but more focal (posterior predominant) in non-E4 carriers (Slattery et al., 2017). In contrast, while WMH was not different between E4 and non-E4 AD patients in one study, WMH volume was associated with worse cognitive performance in all cognitive domains in E4 carriers only with AD or Lewy Body dementia (Mirza et al., 2019). On the other hand, in a combined cohort from cognitively healthy to probable AD patients, E4 homozygotes showed more WMH accumulation per year than non-E4 carriers (Sudre et al., 2017). In addition, in prodromal AD patients, E4 carriers had higher global, temporal, and occipital WMH and poorer memory than non -E4 carriers and E4 strengthened the inverse relationship between WMH and episodic memory (Kumar et al., 2022).

The relationship between WMH and risk of disease and biomarkers of disease seems apoE isoform-dependent. In a meta-analysis, E4 carrier status and E4 homozygosity was associated with increasing WMH and cerebral microbleeds (Schilling et al., 2013). In the same analyses, E2 carrier status was associated with increasing WMH and risk to develop a brain infarct (Schilling et al., 2013).

Amyloid β (Aβ) plaques (Pietroboni et al., 2020) are a hallmark of AD pathology. The AD biomarker tau, not Aβ, is associated with changes in anterior temporal WM integrity (Strain et al., 2018). Remarkably, while WMH is associated with axonal damage, tau independently contributed to the model and decline in white matter is associated with early tau accumulation (Strain et al., 2018).

In E4/E4 carriers with MCI or AD, there was a negative association between WMH and cerebrospinal levels of Aβ42 (Sharms et al., 2022). In contrast, in E3/E3 carriers, WMH was associated with lower levels of total and phosphorylated tau (Sharms et al., 2022). In E2 carriers, including those with MCI, more WMH is seen (Sharms et al., 2022). E2 carriers, like E4 carriers, show an association with WMH but, compared to E3, E2 reduces while E4 increases AD risk. These results suggest that risk to develop AD and to develop cognitive impairments due to WMH are two distinct phenomena. Consistent with this notion, severe WMH is associated with profound cognitive impairments in conditions that like seen in cerebral autosomal dominant arteriopathy with subcortical infarcts and leukoencephalopathy (CADASIL), a rare monogenic cerebral small vessel disease (Jouvent et al., 2020), that is not related to AD (Jolly et al., 2022). Especially in AD, apoE isoform-dependent pathologies related to cortical Aβ and hyperphosphorylated tau might be important to develop cognitive injury that is additive to that resulting from white matter injury alone.

**FIGURE 1:**
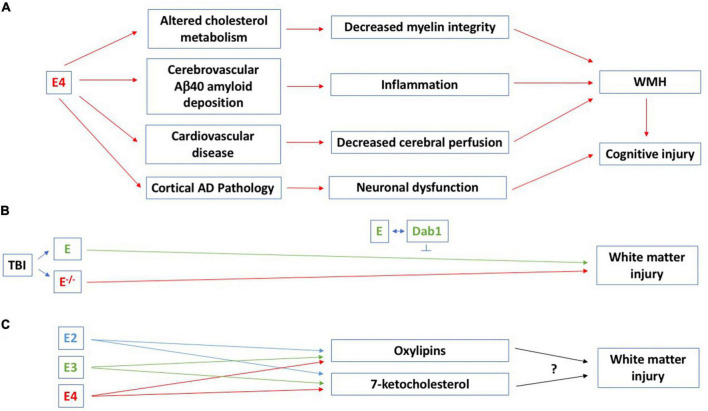
**(A)** Potential pathways involved in WMH and cognitive injury in E4 carriers. All comparisons are to E3/E3 carriers. In cognitively healthy adults (45–75 years of age), pathological WMH is seen in E4 homozygotes (Rojas et al., 2017; Operto et al., 2018) and in participants of the United Kingdom Biobank (mean age: 62 years of age), there is a greater WMH burden in E3/E4 and E4/E4 than E3/E3 carriers. WMH also increased more over a 3-year period in E4 than non-E4 carriers (Cox et al., 2017). E4 affects cholesterol metabolism and DTI radial diffusivity seems especially increased in cognitively healthy E4/E4 carriers, suggesting a disruption in the myelin sheath (Operto et al., 2018). In E4/E4 carriers with MCI or AD, there is a negative association between WMH and cerebrospinal levels of Aβ42 (Sharms et al., 2022). E4 is a risk factor for cardiovascular disease (Li et al., 2021) and effects on WMH and cognitive injury might involve the cardiovascular system and associated inflammation as well. Atherosclerosis and atrial fibrillation are associated with more WMH and this might involve decreased cerebral perfusion (Emrani et al., 2020). E4 is associated with pathological processes possibly linked to WMH and cognitive injury, including Aβ and tau pathologies. E4 also causes increased mitochondrial Ca^2+^ and reactive oxygen species (ROS) levels, mitochondrial dysfunction, and neurotoxicity (Pires and Rego, 2023), which might mediate this effect. **(B)** A role for apoE in white matter integrity in the controlled cortical impact model of traumatic brain injury was revealed 28 days following traumatic injury. The beneficial effects of apoE on white matter integrity involve intracellular adaptor protein Disabled-1 (Dab1) (Abadesco et al., 2014). **(C)** The effects of distinct human apoE isoforms on white matter integrity might be mediated by oxylipins and 7-ketocholesterol. In cognitively healthy hypertensive study participants, docosahexaenoic acid oxylipins are associated with white matter integrity (Silbert et al., 2020). *APOE* genotype modifies the plasma oxylipin response to Omega-3 Polyunsaturated Fatty Acid [eicosapentaenoic acid (EPA) and docosahexaenoic acid (DHA)] supplementation in cognitively healthy people, with E4 carriers showing the greatest increase in plasma oxylipin levels 12 months following the diet supplement (Saleh et al., 2021). For more details, see main text.

White matter capillaries might play an important role in WMH. White matter capillary width is increased in AD, dementia with Lewy bodies, Parkinson’s disease with dementia, vascular dementia, mixed dementias, and post-stroke dementia (Hase et al., 2019). This was suggested to represent a compensatory change to retain white matter perfusion under hypoperfusion conditions (Hase et al., 2019). WMH and cognitive impairments are core features of CAA (Charidimou et al., 2022). In CAA, E4 carriers are at greater risk of Aβ40 deposition in the vasculature, while E2 carriers are at increased risk for vessel breakdown and subsequent cerebral hemorrhage (Greenberg et al., 2020). Amyloid related imaging abnormalities (ARIA) in the setting of anti-amyloid monoclonal antibody therapy for AD consist of punctate WMH (ARIA-E) and often co-localized cerebral microhemorrhages (ARIA-H) (Sperling et al., 2011). The most significant risk factor for ARIA-E in those participating in monoclonal antibody therapeutic trials is E4 carrier status (Sperling et al., 2011; Sevigny et al., 2016; van Dyck et al., 2023). Combined, these findings indicate a critical role of apoE isoform on vascular amyloid deposition and risk for related WMH and hemorrhage in both spontaneous CAA and therapeutic interventions that lead to greater amyloid deposition within the vasculature. Figure 1A illustrates potential pathways involved in WMH and cognitive injury in E4 carriers.

### 1.2. E4 and WMH in mice

To study E4 and WMH in mice, a bilateral carotid artery stenosis model of cerebral hypoperfusion was used in mice with targeted replacement (TR) of E3 or E4. In this mouse model Dr. Sullivan generated (Sullivan et al., 1997; Knouff et al., 1999), human apoE is expressed under control of a mouse apoE promoter. White matter blood flow, local hypoxia and white matter injury were more severely affected in E4 mice than E3 or wild-type mice (Koizumu et al., 2018). In addition, hippocampus-dependent spontaneous alternation in the Y maze and object recognition involving 24 hour delay between learning and memory testing were more severely affected in E4 mice than E3 or wild-type mice (Koizumu et al., 2018), supporting the likely relationship between WMH and cognition. Comparing E4 to E3 or wild-type mice, the following results were observed: (1) More demyelination in the corpus collosum; (2) more reduced levels of the myelin-associated glycoprotein; (3) a higher ratio of myelin basic protein to the axonal marker SM1312; and (4) more reduced levels of the oligodendrocyte marker Olig2. Consistent with these findings, a more profound loss of integrity of the nodes of Ranvier were observed in a model of cerebral hypoperfusion (Koizumu et al., 2018).

### 1.3. ApoE and white matter integrity

Studies in mice lacking apoE support a role for apoE in white matter integrity following in the controlled cortical impact model of traumatic brain injury. ApoE-deficient mice showed impaired white matter integrity, analyzed as reduced fractional anisotropy levels, 28 days following traumatic injury and this was associated with cognitive injury (Huang et al., 2020). This effect was delayed, as no genotype difference in fractional anisotropy was seen three days following traumatic brain injury. The beneficial effects of apoE on white matter integrity involve intracellular adaptor protein Disabled-1 (Dab1), which is normally phosphorylated when apoE binds the very-low density lipoprotein receptor and the apoE receptor 2 (Abadesco et al., 2014; Figure 1B). Reduced fractional anisotropy is also seen in completely denervated nerve segments compared to uninjured sciatic nerve and restored toward normal in regenerating nerve segments (Lehman et al., 2010) and associated with cognitive performance in the water maze test in mice 2 months following hypoxia-ischemia at day 9 (Cengiz et al., 2011).

### 1.4 ApoE, oxylipins, 7-ketocholesterol, and white matter integrity

Effects of distinct human apoE isoforms on white matter integrity might be mediated by oxylipins and 7-ketocholesterol (Figure 1C). Oxylipins are a class of bioactive lipid mediators derived from the oxidation of long-chain polyunsaturated fatty acids (PUFAs). They act as modulators of vascular tone and inflammation and are potential therapeutic targets in AD and related dementias (for a review, see Shinto et al., 2022). In cognitively healthy hypertensive study participants, docosahexaenoic acid oxylipins are associated with white matter integrity (Silbert et al., 2020). *APOE* genotype modifies the plasma oxylipin response to Omega-3 Polyunsaturated Fatty Acid [eicosapentaenoic acid (EPA) and docosahexaenoic acid (DHA)] supplementation in cognitively healthy people, with E4 carriers showing the greatest increase in plasma oxylipin levels 12 months following the diet supplement (Saleh et al., 2021).

Oxysterols are oxidized metabolites of cholesterol. They can penetrate the blood-brain barrier and modulate cholesterol metabolism in the brain (Björkhem, 2013). Levels of the oxysterol 7-ketocholesterol increase with disease progression in the frontal cortex of AD patients (Testa et al., 2016). Levels of 7-ketocholesterol in the cerebrospinal fluid are associated with white matter integrity in cognitively healthy people (Iriondo et al., 2020); higher levels of 7-ketocholesterol are associated with lower fractional anisotropy (Iriondo et al., 2020). In apoE TR mice, cortical levels of 7-ketocholesterol were increased following chronic variable stress, an animal model of post-traumatic stress disorder, in E4, but not E2 or E3, mice (Torres et al., 2022). Chronic variable stress increased 7-ketocholesterol levels in the liver of E2, E3, and E4 mice (Torres et al., 2022). In addition, independent of chronic variable stress, 7-ketocholesterol levels in plasma were much higher in E2 than E3 or E4 mice (Torres et al., 2022).

## 2. Conclusion

WMH is associated with cognitive injury in various neurological conditions. We hypothesize that apoE plays a critical role in modulating WMH via apoE isoform-dependent effects on oxylipins and 7-ketocholesterol, as well as amyloid related vascular injury, as seen in CAA (Figure 1). We recognize that as there are relatively few reported studies regarding some of the topics we discussed in this review. Therefore, future studies are warranted to determine the pathways involved in the relationship between apoE isoform, WMH, and cognitive injury.

## Author contributions

Both authors have drafted, edit, and agreed to the published version of the manuscript.
